# CBX2 enhances the progression and TMZ chemoresistance of glioma via EZH2-mediated epigenetic silencing of PTEN expression

**DOI:** 10.3389/fphar.2024.1430891

**Published:** 2024-07-24

**Authors:** Jian Wang, Bo Yang, Yingzhao Wang, Shuhan Liu, Changkai Ma, Jianmin Piao, Shiqiang Ma, Dehai Yu, Wei Wu

**Affiliations:** ^1^ Department of Neurovascular Surgery, The First Hospital of Jilin University, Changchun, China; ^2^ Department of Thoracic Surgery, The First Hospital of Jilin University, Changchun, China; ^3^ Department of Neurology, Qianwei Hospital of Jilin Province, Changchun, China; ^4^ Core Facility, The First Hospital of Jilin University, Changchun, China

**Keywords:** CBX2, glioma, temozolomide, EZH2, PTEN

## Abstract

Chromobox (CBX) 2, a member of the CBX protein family and a crucial component of the polycomb repressive complex (PRC), exerts significant influence on the epigenetic regulation of tumorigenesis, including glioma. However, the precise role of CBX2 in glioma has remained elusive. In our study, we observed a substantial upregulation of CBX2 expression in glioma, which displayed a strong correlation with pathological grade, chemoresistance, and unfavorable prognosis. Through a series of *in vivo* and *in vitro* experiments, we established that heightened CBX2 expression facilitated glioma cell proliferation and bolstered resistance to chemotherapy. Conversely, CBX2 knockdown led to a significant inhibition of glioma cell growth and a reduction in chemoresistance. Notably, our investigation uncovered the underlying mechanism by which CBX2 operates, primarily by inhibiting PTEN transcription and activating the AKT/mTOR signalling pathway. Conversely, silencing CBX2 curtailed cell proliferation and attenuated chemoresistance by impeding the activation of the PTEN/AKT/mTOR signalling pathway. Delving deeper into the molecular intricacies, we discovered that CBX2 can recruit EZH2 and modulate the trimethylation of histone H3 lysine 27 (H3K27me3) levels on the PTEN promoter, effectively suppressing PTEN transcription. Our research unveils a comprehensive understanding of how CBX2 impacts the tumorigenesis, progression, chemoresistance, and prognosis of glioma. Furthermore, it presents CBX2 as a promising therapeutic target for drug development and clinical management of glioma.

## Introduction

Glioma is the most common malignant primary brain tumour originating from glial cells, accounting for nearly one-third of the total tumours of the central nervous system and four-fifths of all malignant tumours of the central nervous system (Ostrom et al., 2015). The characteristics of glioma include invasive growth, rapid progression, chemoresistance, difficult to complete resection, frequent recurrence and poor prognosis. According to World Health Organization (WHO) criteria, low-grade glioma (LGG) include WHOI/II, while high-grade glioma (HGG) includes WHO III/IV gliomas. Most WHO IV grade gliomas are glioblastoma (GBM), which is the most fatal type of glioma and easily recurs (Louis et al., 2016). Standard treatments for glioma include surgical resection, radiotherapy, chemotherapy, targeted therapy and supportive therapy. Although these therapeutic approaches have been improved in the clinic, GBM patient prognosis is still poor, and the median survival time of GBM patients is less than 2 years (Wen and Kesari, 2008; Yang et al., 2022). Multiple studies have reported that the tumorigenesis, progression, recurrence and chemoresistance of glioma are closely related to the abnormal overexpression of certain oncogenes and the loss or low expression of certain tumour suppressor genes (Chen et al., 2013; Yao et al., 2016; Ganguly et al., 2018; Zhang et al., 2019). Therefore, a deep understanding of the molecular mechanism of glioma is crucial for accurately predicting its occurrence and development.

Polycomb group (PcG) complexes silence tumour suppressor genes [e.g., *p16* (Kotake et al., 2015), *Rap1GAP* (Banerjee et al., 2012) and *DOK1* (Siouda et al., 2014)] through the modification of histones, which play an important role in the regulation of tumorigenesis and progression (Chan and Morey, 2019). There are two subtypes of PcG complexes, polycomb repressive complex (PRC) 1 and PRC2 (Shao et al., 1999; Levine et al., 2002). The chromobox (CBX) protein family is a key component of PcG-mediated gene repression. CBX2 a 56 kDa protein, is encoded by the *CBX2* gene, which is located in q25.3 of chromosome 17 (Gecz et al., 1995). It is a member of the CBX protein family and a component of PRC1. CBX2 is dysfunctional in many cancers and plays a key role in epigenetic regulation of cancer development and growth-related genes. For example, Hu et al. (2022) reported that CBX2 downregulates tumour suppressor genes (*PPARG*, *OLR1* and *FABP5*) through epigenetic modification of the promoter region to promote the growth and metastasis of lung adenocarcinoma. As the overexpression of CBX2 is closely correlated with a poor prognosis for tumours (Wheeler et al., 2018; Zeng et al., 2021; Del Gaudio et al., 2022), CBX2 is expected to be a potential promising therapeutic target for cancer gene therapy. However, the cellular function and the underlying molecular mechanisms of CBX2 in gliomas are still unknown.

In this study, we identified that CBX2 was significantly upregulated in glioma through comprehensive analysis of transcriptomic data from the Cancer Genome Atlas (TCGA) and Chinese Glioma Genome Atlas (CGGA) databases. Furthermore, the expression level of CBX2 is correlated with higher glioma pathological stage and shorter overall survival time in glioma patients. Immunohistochemical (IHC) analysis showed that a high expression level of CBX2 predicts a poorer prognosis in glioma patients. Both *in vivo* and *in vitro* experiments confirmed that overexpression of CBX2 promoted glioma proliferation and chemoresistance. However, knockdown of CBX2 reversed these effects in glioma. Additionally, our experiments confirmed that CBX2 inhibits the expression of PTEN through recruitment of EZH2 and increases the trimethylation of histone H3 lysine 27 (H3K27me3) level of the PTEN promoter, thus activating the AKT/mTOR pathway.

## Materials and methods

### Bioinformatics analysis of gene expression and its prognostic value

To investigate the relationship between CBX2 expression level and the clinicopathological characteristics, including PIK3CA mutation, as well as the potential prognostic value of CBX2 in glioma patients, we downloaded normal brain tissue gene expression data from Genotype-Tissue Expression (GTEx) database [cohort: TCGA TARGET GTEx (n = 19,109)] and glioma tissue gene expression data and the corresponding clinical data [cohort: GDC TCGA Lower Grade Glioma (n = 533), cohort: GDC TCGA Glioblastoma (n = 613)] from TCGA using the UCSC Xena browser (https://xenabrowser.net) (Hu et al., 2020; Zou et al., 2023). R software (version 3.6.3) was used for data collation, analysis and result visualization. Data [DataSet ID: mRNAseq_693 (n = 693), mRNAseq_325 (n = 325), mRNA sequencing (non-glioma as control) (n = 20)] downloaded from CGGA (http://www.cgga.org.cn) were used to verify the results from GTEx and TCGA analysis (Zhao et al., 2021).

### Clinical samples and ethical approval

This study was approved by the Ethics Committee of the First Hospital of Jilin University (Changchun, China) (Approval number: 2022–109), and all participants provided written informed consent. 142 paraffin sections of gliomas were collected from glioma patients who underwent surgery in the First Hospital of Jilin University from January 2012 to December 2018. The clinical data of these patients are shown in Table 1. A COX proportional hazard model was used to analyse the factors related to the overall survival of patients (Table 2). The tumour response to Temozolomide (TMZ) was evaluated according to the Response Evaluation Criteria in Solid Tumours version 1.0 criteria (Bogaerts et al., 2009). Patients with incomplete tumour resection were reported to have complete/partial response (CR/PR) and stable/progressive disease (SD/PD) according to tumour measurements confirmed through repeat studies performed ≥ 4 weeks after the initial fulfilment of the response criteria.

**TABLE 1:** Correlation of the CBX2 expression with clinical and pathological characteristics in tissue samples from glioma patients.

Characteristics	CBX2 expression		Total (n = 142)	*χ2*	*p*-value
	High (n = 74)	Low (n = 68)			
Sex					
Male	38	30	68	0.743	0.389
Female	36	38	74		
Age					
<50	47	52	99	2.818	0.093
≥50	27	16	43		
Location					
Supratentorial	72	62	134	2.497	0.114
Subtentorial	2	6	8		
Histological grade					
I-II	20	54	74	38.97	<0.001*
III-IV	54	14	68		
Tumor size (mm)					
<40	16	33	49	11.35	<0.001*
≥40	58	35	93		
Postoperative recurrence					
Yes	47	30	77	5.371	0.021*
No	27	38	65		
Complete resection					
Yes	51	51	102	0.648	0.421
No	23	17	40		

*p < 0.05 was considered to be statically significant.

**TABLE 2:** COX regression analyses on risk factors influencing the prognosis of glioma.

Characteristics	Univariate analysis			Multivariate analysis		
	HR	95% CI	*p*-value	HR	95% CI	*p*-value
Age (years)						
<50 vs. ≥50	0.924	0.626–1.365	0.693			
Gender						
Male vs. Female	1.118	0.783–1.595	0.54			
Location						
Supratentorial vs. Subtentorial	1.596	0.701–3.635	0.265			
Tumor size (mm)						
≥40 vs. <40	2.082	1.404–3.088	<0.001*	1.228	0.804–1.876	0.342
Histological grade						
III + IV vs I + II	4.549	3.049–6.788	<0.001*	2.513	1.552–4.071	<0.001*
Complete resection						
Yes vs. No	0.925	0.623–1.375	0.7			
Postoperative recurrence						
Yes vs. No	3.096	2.127–4.506	<0.001*	2.166	1.445–3.249	<0.001*
CBX2 expression						
High vs. Low	2.958	2.033–4.302	<0.001*	1.656	1.049–2.612	0.03*

*p < 0.05 was considered to be statically significant.

### IHC staining and evaluation

The paraffin-embedded tissues were cut into 4 μm slices and stained with IHC according to the standard procedure. The slices were dewaxed and hydrated and sealed with 3% H_2_O_2_ for 10 min. The slices were then placed in 0.1% citric acid buffer of pH 6.0 for hyperbaric heating antigenic repair for 20 min and finally incubated with anti-CBX2 primary antibody (1:200, ab235305, Abcam, United States) overnight at 4°C. The next day, the slices were restored to room temperature, and each slice was incubated with 25 μL of HRP-labelled secondary antibody in an immunohistochemistry kit (D601037, Sangon, China) at 37°C for 30 min. DAB staining, haematoxylin restaining and gradient alcohol dehydration were performed. After observing and taking images under an optical microscope (Olympus, Japan), two pathologists blinded to the patients’ data implemented the immunoreactive score (IRS) system to evaluate the expression level of CBX2 (Liu et al., 2021).

The IRS formula was as follows: IRS = percentage of positive cells (PP) × staining intensity (SI). PP was scored as follows: 0 points for <5%; 1 point for 5%–25%; 2 points for 26%–50%; 3 points for 51%–75%; and 4 points for 75%–100%. The SI was determined as follows: 0 points, no staining; 1 point, weak staining (light yellow); 2 points, moderate staining (yellow brown); and 3 points, strong staining (brown). IRS values between 0–4 and 5–12 represented low protein expression and high protein expression, respectively.

### Cell lines and culture conditions

Human glioma cell lines (A172, LN229 and T98G) were purchased from Procell Life Science and Technology (China). The human glioma cell line LN18 was purchased from JENNIO Biological Technology (China). The healthy glial cell line HEB was purchased from BeNa Culture Collection (China). All these cells were cultured in Dulbecco’s modified Eagle’s medium (DMEM) (PM150210, Procell, China) supplemented with 10% foetal bovine serum (FBS) (FSP 500, ExCell Bio, China), 100 units/mL penicillin and 100 μg/mL streptomycin (PB180120, Procell) at 37°C with 5% CO2. All cell lines were authenticated by STR profiling and underwent *mycoplasma* testing to confirm their absence of contamination by *mycoplasma*.

### Establishment of TMZ resistant cells

The establishment of TMZ resistant cells in LN229 cells followed a protocol outlined in Wu’s prior studies (Wu et al., 2019). In brief, TMZ was introduced to the cell culture medium at an IC50 1/50 concentration (1.8 μM) for cultivating LN229 cells in six-well plates. Once the cells achieved stable growth, the drug dosage was increased in multiples. Each dosage level was maintained for a duration of 15 days, extending through the course of 5 months. The induced TMZ-resistant cells were designated as “LN229R.”

### Construction of plasmids, transfection and establishment of stable cell lines

Lentivirus was used for gene overexpression and knockdown. For overexpression of CBX2, human CBX2 cDNA was synthesized and cloned into pGreen.Neo vector to generate pGreen.Neo-CBX2 recombinant plasmid. To knockdown CBX2, short hairpin RNAs (shRNAs) targeting CBX2 mRNA [shCBX2-0 (Rhodes et al., 2004), shCBX2-1 (Rhodes et al., 2004), shCBX2-2] were cloned into pGreen.Puro vector to generate pGreen.Puro-shCBX2 recombinant plasmids. The sequences are shown in Supplementary Table S1.

For lentiviral transfection, a total of 2 × 10^5^ 293 T cells were seeded in 6-well plates with antibiotic-free complete medium and cultured for 24 h, and then recombinant plasmid and packaging plasmids pSPAX2 and pMD2.G were cotransfected into cells to package lentivirus using Lipofectamine 2,000 Transfection Reagent (11668–019, Invitrogen, United States) according to the manufacturer’s manual. The virus-containing supernatant was harvested at 24 and 48 h posttransfection. Stable cell lines were established by infection of LN229, LN229R and LN18 cells with lentivirus solution and selection with puromycin (1 μg/mL, ST551, Beyotime, China) or G418 sulfate (600 μg/mL, A600958, Sangon).

### Drugs

Dimethyl sulfoxide (DMSO, D4540, Sigma, United States) was used to dissolve TMZ powder (HY-17364, MedChem Express, China), MK2206 powder (SF2712, Beyotime) and GSK126 powder (SC0060, Beyotime) to final concentrations of 50, 10, and 10 mM, respectively. To evaluate the effect of CBX2 on glioma chemosensitivity, we treated glioma cells with a series of doses of TMZ (0, 15.625, 31.25, 62.5, 125, 250, 500, 1,000 μM) for 48 h, and cell viability was determined using a CCK-8 assay. Data are displayed as a percentage normalized to the viability of cells with no TMZ treatment. The abscissa is the logarithm of TMZ concentration (log conc.). IC50 represents the concentration of TMZ that reduced cell viability to 50%.

### CCK-8 assay

Cell viability was measured using the Cell Counting Kit-8 (CCK-8) method. Briefly, the transfected cells were seeded into one well of a 96-well plate at a density of 3,000 cells/well. After 24, 48, 72, and 96 h, 10 μL of CCK-8 solution (40203ES80, Yeasen, China) was added to 100 μL media of each well and incubated for 1 h at 37°C in the dark. The absorbance was measured at 450 nm by applying a microplate reader (Bio Tek, United States).

### Colony formation assay

The transfected glioma cells were seeded in 6-well plates at a density of 1,000 cells/well for the colony formation assay. After the cells were cultured for 14 days, visible colonies were fixed with paraformaldehyde (4% w/v) for 30 min and stained with crystal violet (0.1% w/v) for an additional 1 h. Each cell group was cultured in triplicate, and surviving colonies (>50 cells/colony) were counted under a microscope (Olympus).

### EdU assay

EdU assay was performed with an EdU Cell Proliferation Kit with Alexa Fluor 555 (C0075, Beyotime) according to the manufacturer’s instructions. After staining, the cells were observed with an inverted fluorescence microscope (Olympus). The images were processed and analysed by ImageJ software (version 1.8.0). The percentage of EdU-positive cells in the total number of cells in each visual field was measured.

### Quantitative real-time PCR (qRT-PCR)

Total RNA was extracted from cultured cells by applying an Easy Pure RNA Kit (ER101, TransGen, China) according to the manufacturer’s protocol. Then, 1 μg of total RNA was subjected to the synthesis of the first strand cDNA with the TransScript^®^ All-in-One First-Strand cDNA Synthesis Kit (AT341, TransGen). Finally, cDNA fragments were used for qRT-PCR and amplified with FastStart Universal SYBR^®^ Green Master Mix (ROX) (04913914001, Roche, Germany) according to the manufacturer’s instructions. Data were analysed by the 2^−ΔΔCT^ method, and GAPDH was used as an endogenous control. All primers used in this study are shown in Supplementary Table S1.

### Western blotting (WB)

Total protein extracted from cultured cells was subjected to isolation using lysis buffer (P0013, Beyotime) mixed with protease inhibitor cocktail (P1045, Beyotime), followed by being separated through sodium dodecyl sulfate-polyacrylamide gel electrophoresis (SDS-PAGE). Subsequently, the samples were transferred to polyvinylidene fluoride (PVDF) membranes and blocked in protein free rapid blocking buffer (EpiZyme, PS108). The membranes were incubated with primary antibodies at 4°C overnight. After being washed in TBST, the samples were incubated with the secondary antibodies. Finally, the results were visualized by enhanced chemiluminescence substrate. The relevant information regarding the antibodies is summarized in Supplementary Table S2.

### Flow cytometric analysis

Cell apoptosis was analysed using the PE Annexin V Apoptosis Detection Kit I (BD Biosciences, United States), and the apoptosis rate was evaluated by fluorescence-activated cell sorting (FACS). The cells in the different portions represented the different cell states as follows: the necrotic cells were present in the upper left portion Q1: Annexin V-PE(−)/7-AAD(+), the late-apoptotic cells were present in the upper right portion Q2: Annexin V-PE(+)/7-AAD(+), the early apoptotic were cells present in the lower right portion Q3: Annexin V-PE(+)/7-AAD(−) and the viable cells were present in the lower left portion Q4: Annexin V-PE(−)/7-AAD(−). Apoptosis rate = Q2 + Q3.

### Coimmunoprecipitation (co-IP)

For co-IP, according to the IP assay kit (P2179, Beyotime) manufacturer’s protocol, the cells were lysed on ice with lysis buffer and protease inhibitor cocktail. A certain amount of cleavage product was left as an input control. The remaining products were divided into three parts and incubated with anti-CBX2 antibody, anti-EZH2 antibody and normal rabbit IgG (A7016, Beyotime) overnight at 4°C. On the second day, the protein A/G magnetic beads were added and incubated at room temperature for 1 h. After incubation, the beads were placed on the magnetic frame for 10 s for separation, and the supernatant was removed. The products were then washed with lysis buffer, collected and analysed by WB. The information of antibodies is summarized in Supplementary Table S2.

### Chromatin immunoprecipitation (ChIP) assay

During the preliminary experiment, we strategically devised 9 predicted binding sites that encompassed the entirety of the *PTEN* promoter region and exon 1, spanning from −2,000 bp to + 1,000 bp. Within the first 7 sites, positioned before the transcriptional start site (TSS), we identified and chose the two sites with the strongest binding affinity for further investigation, which we designated as “site1” and “site2.” However, among the 2 sites located within exon 1, there was no apparent evidence of binding. In light of this, we opted to select one of these sites as a representative, denoted as “site3.” ChIP assays were performed with a ChIP assay kit (P2078, Beyotime) based on the manufacturer’s protocol. Briefly, glioma cells were collected and fixed with 1% formaldehyde solution for 10 min at 37°C and quenched with 0.125 M glycine for 5 min. DNA fragments ranging from 200 bp to 1,000 bp were generated using sonication and then pulled down by anti-CBX2 antibody, anti-EZH2 antibody and anti-H3K27me3 antibody, respectively. The crosslinked molecules were reversed, and DNA was purified. Finally, the precipitated DNA was analysed by qPCR. Normal rabbit IgG (A7016, Beyotime) was used as the negative control. The information of primers and antibodies are listed in Supplementary Tables S1, S2, respectively.

### Animal study

All 6-week-old male high immunodeficiency model-NCG mice were purchased from Gempharmatech Co., Ltd. (China) and maintained in special pathogen-free conditions. The mice were randomized to assign groups using random number table method after adapting to the feeding environment for 1 week. Intracranial xenografts were implanted as Zeng and his colleagues described (Zeng et al., 2017). To assess chemoresistance, mice were intraperitoneally injected with 20 mg/kg TMZ followed by a standard schedule of 4 weeks on and 4 weeks off treatment from the second week after tumor inoculation (recorded as 0 days). We performed IVIS imaging of intracranial tumors at 7, 14, 21, and 28 days to observe tumor progression, and this process was carried out without blinding. In addition, We sacrificed a separate cohort of mice 5 weeks after tumor inoculation for WB (n = 3). The mouse brains were harvested and proteins were extracted. All animal manipulations were performed in accordance with the ethics guidelines of the First Hospital of Jilin University (Approval number: 20210728).

### Statistical analysis

All experiments were repeated independently at least three times. The count data were analysed by the chi-squared (χ^2^) test. The Student’s unpaired *t*-test (for data following a normal distribution) or non-parametric Mann-Whitney U test (for data not following a normal distribution) was used to analyse significant differences between two groups. For comparisons between multiple groups, one-way ANOVA was applied. Kaplan-Meier survival analyses were conducted to estimate overall survival using the log-rank test. A *p*-value of <0.05 was considered significant. The statistical analyses were performed using GraphPad Prism 9.2.0 or SPSS 23.0 software.

## Results

### CBX2 was upregulated in glioma and related to glioma grade, TMZ chemoresistance and a poor prognosis

**FIGURE 1:**
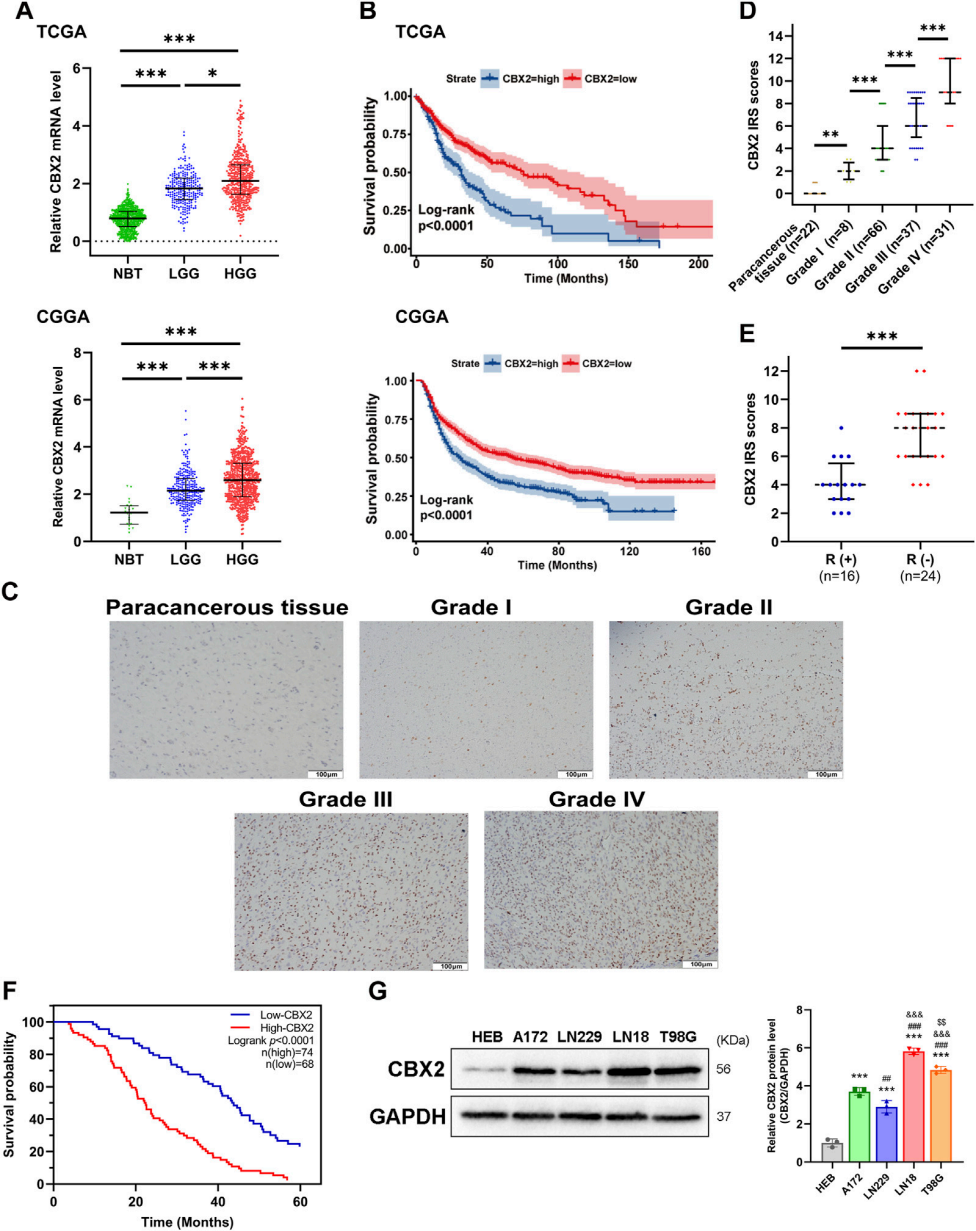
CBX2 is upregulated in glioma and related to tumour grade, TMZ chemoresistance and a poor prognosis. **(A)** CBX2 mRNA expression analysis in glioma (LGG and GBM) versus NBT by using TCGA, GTEx and CGGA databases (*p < 0.05, ***p < 0.001). **(B)** Kaplan–Meier overall survival analysis for CBX2 mRNA expression by using TCGA and CGGA databases (*p* < 0.0001). **(C)** Representative IHC images of CBX2 in paracancerous tissue samples and grade I-IV glioma samples. **(D)** IRS of IHC (**p < 0.01, ***p < 0.001). **(E)** IRS showing response (R+) or no response (R-) to TMZ in glioma samples (***p < 0.001). **(F)** Kaplan-Meier overall survival analysis for CBX2 expression using glioma patient samples (*p* < 0.0001). **(G)** CBX2 levels in HEB and human GBM cell lines (***p < 0.001, compared with HEB; ##*p* < 0.01, ###*p* < 0.001, compared with A172; &&&*p* < 0.001, compared with LN229; $$*p* < 0.01, compared with LN18).

We used TCGA, GTEx and CGGA datasets to analyse the expression level and clinical significance of CBX2 in glioma. The analysis results showed that compared with that in normal brain tissue (NBT), CBX2 expression was higher in both LGG and HGG, with the highest expression in HGG (Figure 1A). Survival curve analysis showed that patients with high levels of CBX2 had a poorer prognosis than those with low levels (Figure 1B). To better understand the clinical significance of CBX2, samples from glioma patients who underwent neurosurgery were stained with IHC, and the IRS value was used to evaluate the expression level of CBX2. We found that the expression level of CBX2 increased significantly with the progression of WHO grade (Figures 1C, D). Among the 40 patients with incomplete resection of tumours, the IRS values of 24 patients with a negative TMZ reaction (SD/PD) were higher than those of 16 patients with a positive reaction (CR/PR) (*p* < 0.001), which indicated that high expression of CBX2 is associated with TMZ chemoresistance (Figure 1E). We analysed the correlation between the expression level of CBX2 and the clinicopathological features of glioma patients. According to the IRS value, the patients were divided into a low expression group (n = 68) and a high expression group (n = 74). The clinicopathological analysis showed that the expression level of CBX2 was significantly correlated with tumour size (*p* < 0.001), histological grade (*p* < 0.001) and postoperative recurrence (*p* < 0.021) (Table 1). Compared with patients with a low level of CBX2, patients with a high level of CBX2 had a larger tumour size (≥40 mm), higher histological grade (grade III-IV) and higher risk of postoperative recurrence. Moreover, univariate and multivariate Cox proportional hazard regression models showed that histological grade (95% CI 1.552–4.071, *p* < 0.001), postoperative recurrence (95% CI 1.445–3.249, *p* < 0.001) and CBX2 level (95% CI 1.049–2.612, *p* = 0.03) significantly affected survival time (Table 2). In addition, the median survival times of patients with high and low levels of CBX2 were 22.4 and 43.6 months, respectively. Kaplan-Meier survival analysis showed that glioma with higher CBX2 levels had shorter overall survival times and worse prognoses than patients with lower CBX2 levels (Figure 1F, *p* < 0.0001). We also detected the CBX2 level in A172, LN18, LN229 and T98G glioma cell lines using WB, and the results showed that compared with normal glial cells (HEB), CBX2 levels were significantly upregulated in all glioma cell lines, especially in LN18 cells (Figure 1G). These data suggest that CBX2 is upregulated in glioma tissues and glioma cell lines, which is related to the malignant phenotype of glioma, TMZ chemoresistance and a poor prognosis in patients.

### Knockdown of CBX2 inhibited cell viability, reduced cell proliferation, and enhanced TMZ chemosensitivity of glioma

**FIGURE 2:**
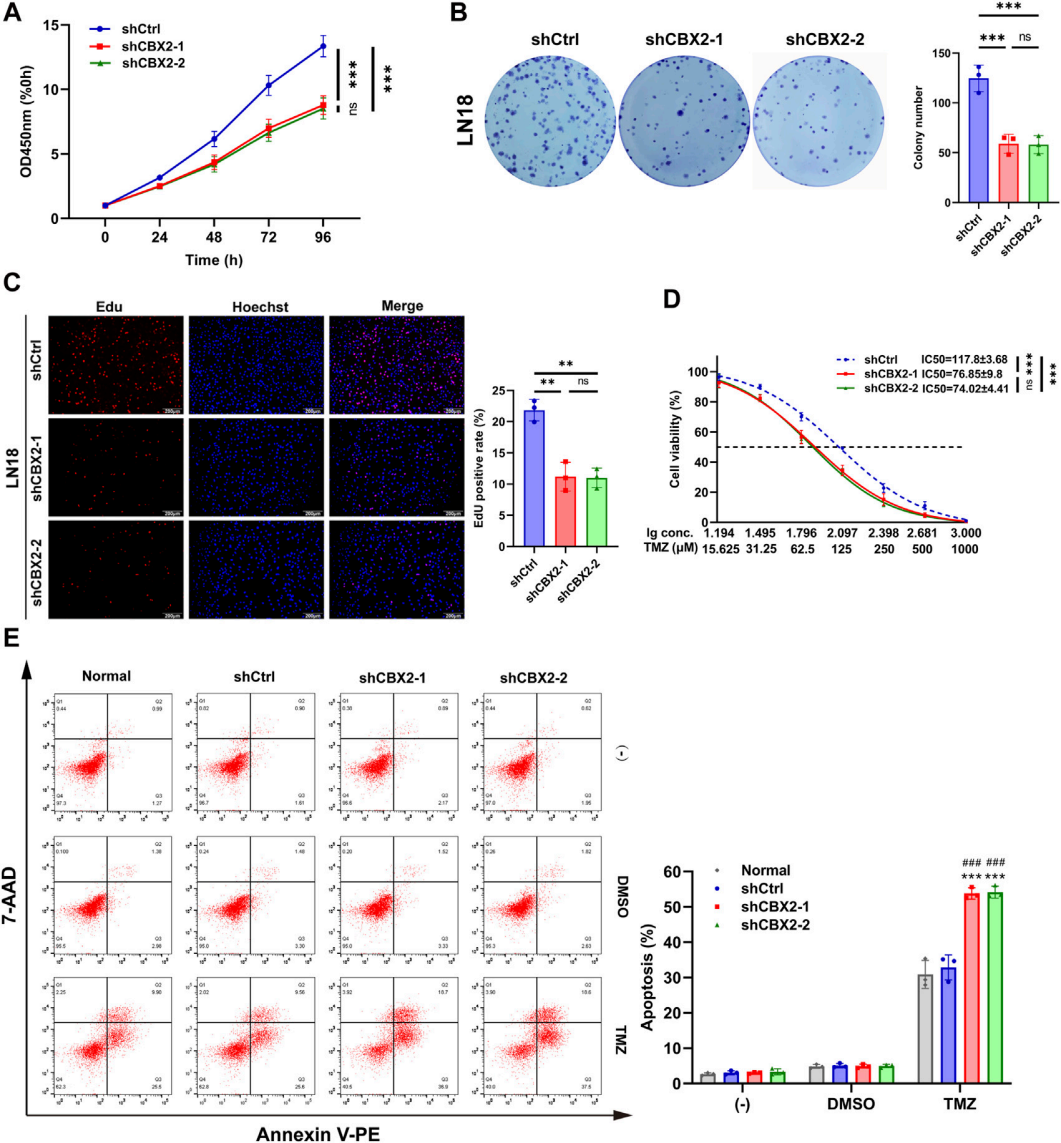
Knockdown of CBX2 inhibited cell viability, reduced cell proliferation, and enhanced TMZ chemosensitivity of glioma. **(A)** CCK-8 assay demonstrated that knockdown of CBX2 inhibited the viability of LN18 cells. **(B,C)** Colony formation and EdU assay demonstrated that knockdown of CBX2 suppressed the proliferation of LN18 cells. **(D)** Transfected LN18 cells were treated with a series of doses of TMZ, and the cell viability was determined using a CCK-8 assay (**p < 0.01, ***p < 0.001; ns: not significant). **(E)** Normal and transfected LN18 cells were treated with 200 μM TMZ for 48 h, and cell apoptosis was assessed using flow cytometry (***p < 0.001, compared with Normal; ###*p* < 0.001, compared with shCtrl).

In order to investigate the role of CBX2 in GBM, we opted to perform a CBX2 knockdown experiment in the LN18 cell line. This choice was motivated by the higher baseline expression of CBX2 in the LN18 cell line, which is expected to yield a more effective knockdown and consequently result in more pronounced alterations in the cellular biological properties (Supplementary Figure S1A). The CCK-8 assay revealed that at 96 h, the OD450 nm value for the ShCtrl group was 13.36 ± 0.82 times that of 0 h, whereas for shCBX2-1 it was 9.79 ± 0.72 and for shCBX2-2 it was 8.52 ± 0.82, indicating significant inhibition of cell viability upon CBX2 knockdown (Figure 2A). The colony number of the shCBX2-1 and shCBX2-2 groups was half that of the shCtrl groups showed that knockdown of CBX2 decreased cell proliferation (Figure 2B). The Edu positive rate of the shCBX2-1 and shCBX2-2 groups was approximately half that of the shCtrl group, confirming that knockdown of CBX2 significantly suppressed cell proliferation (Figure 2C). The IC50 of TMZ in shCtrl is 117.8 ± 3.68 μM, while that in shCBX2-1 and shCBX2-2 are 76.85 ± 9.8 and 74.02 ± 4.41 μM, respectively (Figure 2D). Next, we treated LN18 cells with 200 μM TMZ and detected cell apoptosis. As shown in Figure 2E, knockdown of CBX2 significantly increased the TMZ sensitivity of LN18 cells, apoptosis rate increased from nearly 30% to over 50%. In summary, these data suggest that suppression of CBX2 in glioma cells can inhibit cell viability, reduce cell proliferation and enhance TMZ chemosensitivity.

### Overexpression of CBX2 enhanced cell viability and promoted cell proliferation and TMZ chemoresistance of glioma

**FIGURE 3:**
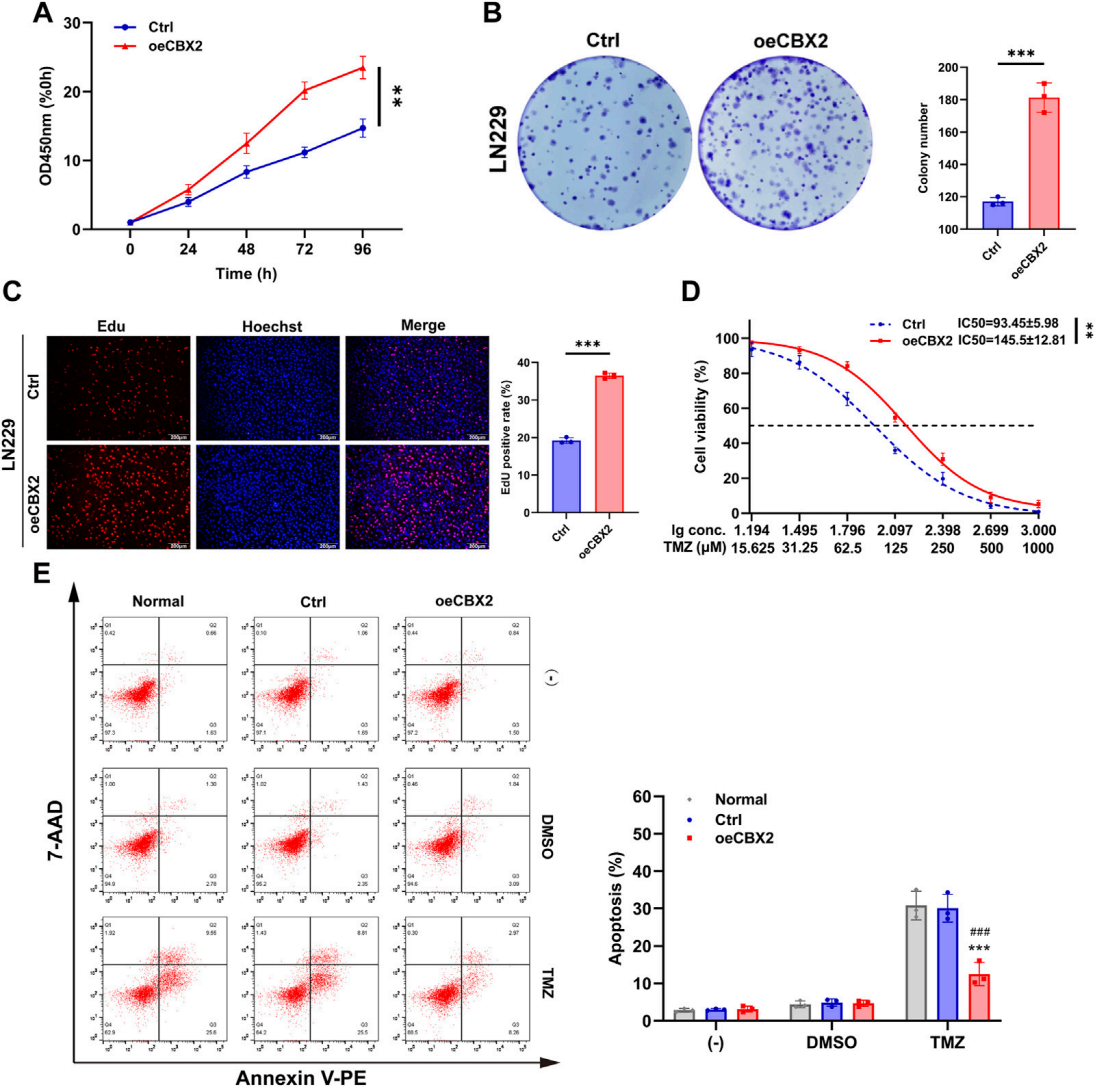
CBX2 overexpression increased cell viability and promoted cell proliferation and chemoresistance of glioma. **(A)** CCK-8 assay demonstrated that CBX2 overexpression enhanced the viability of LN229 cells. **(B,C)** Colony formation and EdU assay demonstrated that CBX2 overexpression promoted the proliferation of LN229 cells. **(D)** Transfected LN229 cells were treated with a series of doses of TMZ, and the cell viability was determined using a CCK-8 assay (**p < 0.01, ***p < 0.001). **(E)** Normal and transfected LN229 cells were treated with 200 μM TMZ for 48 h, and cell apoptosis was assessed using flow cytometry (***p < 0.001, compared with Normal; ###*p* < 0.001, compared with Ctrl).

To further validate the involvement of CBX2 in glioma progression, we introduced exogenous CBX2 into LN229 cells. This choice was driven by the lower baseline expression of CBX2 in the LN229 cell line, which is anticipated to result in a more effective overexpression and consequently bring about more significant alterations in the cellular biological properties (Supplementary Figure S1B). The CCK-8 assay revealed that at 96 h, the OD450 nm value for the Ctrl group was 14.73 ± 1.32 times that of 0 h, whereas for oeCBX2 it was 23.51 ± 1.63 (Figure 3A). The colony number of the oeCBX2 group is 1.55 times that of the Ctrl (Figure 3B). The Edu positive rate in the oeCBX2 group was 1.9 times that of the Ctrl group (Figure 3C). The above results showed that overexpression of CBX2 significantly increased cell viability and promoted cell proliferation. The IC50 of TMZ in Ctrl is 93.45 ± 5.98 μM, while that of oeCBX2 is 145.5 ± 12.81 μM (Figure 3D). Cellular apoptosis analysis showed that CBX2 overexpression reduced TMZ-induced apoptosis from 30% to 12%. The results showed that CBX2 overexpression significantly decreased the TMZ sensitivity of LN229 cells (Figure 3E). These experiments suggested that overexpression of CBX2 can enhance cell viability, promote cell proliferation and enhance TMZ chemoresistance.

### CBX2 promoted cell viability, proliferation and TMZ chemoresistance of glioma by inhibiting PTEN and activating the AKT/mTOR signalling pathway

**FIGURE 4:**
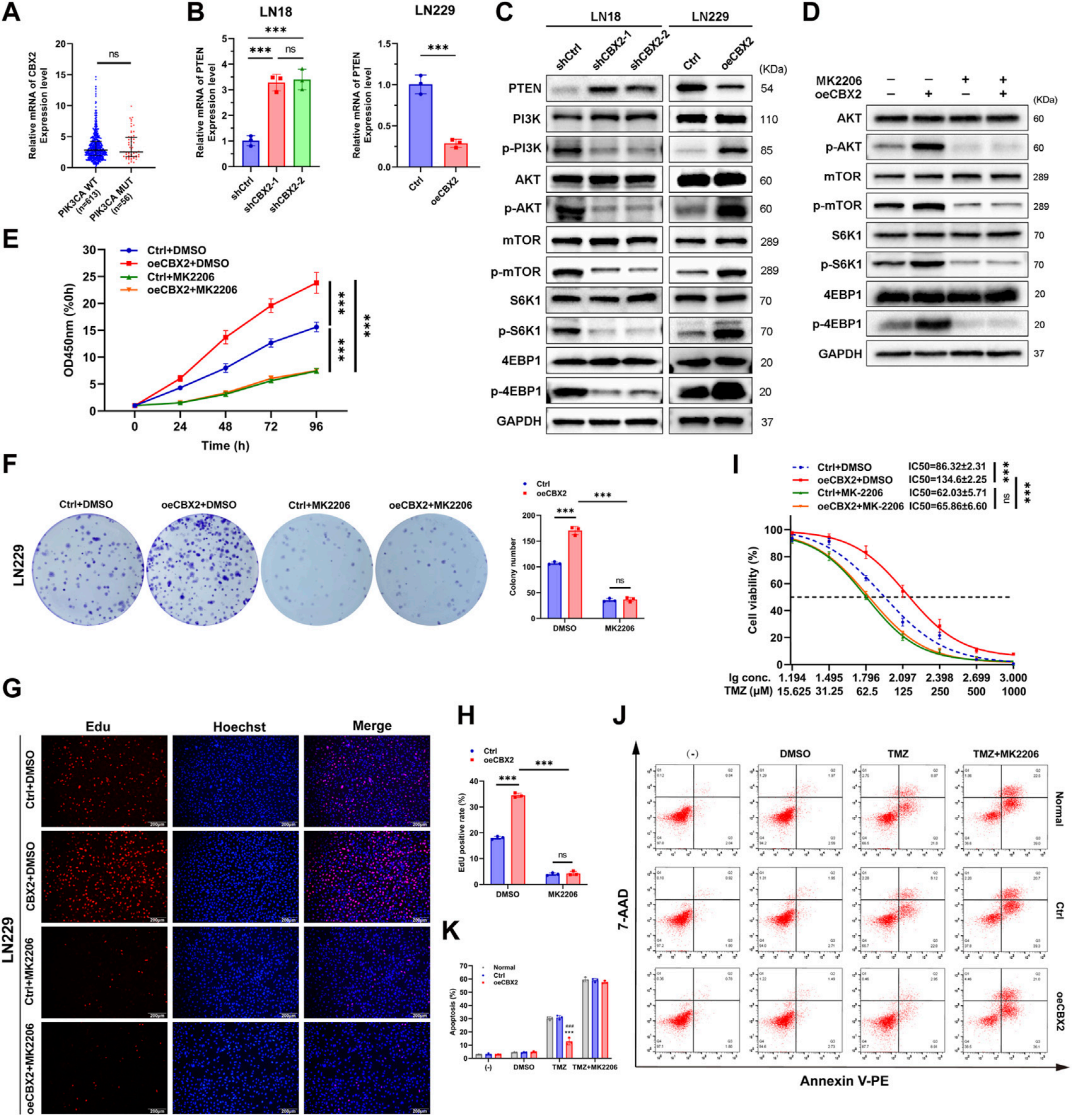
CBX2 promoted cell viability, proliferation and TMZ chemoresistance of glioma by inhibiting PTEN and activating the AKT/mTOR signalling pathway. **(A)** Analysis of TCGA datasets showed that there was no significant difference in CBX2 expression between the PIK3CA WT and PIK3CA MUT cases. **(B)** Knockdown CBX2 increased PTEN mRNA expression in LN18 cells, while overexpression of CBX2 decreased PTEN mRNA expression in LN229 cells (***p < 0.001; ns: not significant). **(C)** PTEN is involved in CBX2-mediated AKT/mTOR activation. LN18 cells transfected with CBX2 shRNAs and LN229 cells transduced with human CBX2 via lentivirus were harvested and subjected to WB to determine the relative protein levels. **(D)** Inhibition of AKT repressed CBX2-mediated activation of the AKT/mTOR signalling pathway. CBX2-overexpression LN229 cells were treated with the AKT inhibitor MK2206 (1 μM) for 24 h, and then the cells were harvested and subjected to WB to determine the relative protein levels. **(E–H)** Inhibition of AKT repressed CBX2-induced cell viability and proliferation. Transfected LN229 cells treated with/without MK2206 were subjected to a CCK-8 assay **(E)**, colony formation assay **(F)**, EdU assay **(G,H)**. **(I)** Inhibition of AKT repressed CBX2-induced TMZ resistance. LN229 cells were treated with different drugs, cell viability was determined using a CCK-8 assay (***p < 0.001; ns: not significant); **(J,K)** Cell apoptosis was assessed using flow cytometry (***p < 0.001, compared with Normal; ###*p* < 0.001, compared with Ctrl).

Previous studies have shown that the AKT/mTOR signalling pathway is involved in the occurrence, development and chemoresistance of gliomas (Murphy et al., 2016; Lo Dico et al., 2019; Tang et al., 2022). CBX2 promotes glioma cell proliferation and invasion through the PI3K/AKT pathway (Wang et al., 2021). Given the prevalence of PIK3CA mutations in glioma tumors, we initiated an analysis of the association between CBX2 and PIK3CA mutations using TCGA datasets. The findings revealed that there was no significant difference in CBX2 expression between the PIK3CA wild-type (WT) and PIK3CA mutant-type (MUT) cases, as depicted in Figure 4A.

PTEN is a key upstream inhibitor of the AKT/mTOR signalling pathway. By inhibiting the phosphorylation of key proteins in the PI3K/AKT/mTOR pathway, PTEN controls numerous cancer cellular processes, including survival, proliferation, energy metabolism and chemoresistance (Li et al., 2016; Nan et al., 2018; Ding et al., 2019; Ashrafizadeh et al., 2020).Therefore, we speculate that there is a certain interaction between CBX2 and PTEN in gliomas, thus indicating that CBX2 regulates the AKT/mTOR pathway.

To substantiate the regulatory connection between CBX2 and PTEN, we assessed alterations in the mRNA and protein expression of PTEN in glioma cells following CBX2 knockdown or overexpression. As shown in Figures 4B, C; Supplementary Figure S1C, we found that knockdown of CBX2 increased the expression of PTEN at both the mRNA and protein levels in LN18 cells. In contrast, the introduction of exogenous CBX2 suppressed the expression of PTEN in LN229 cells. We further confirmed that knockdown of CBX2 decreased the activation of the PI3K/AKT/mTOR signalling pathway in LN18 cells, while overexpression of CBX2 activated the PI3K/AKT/mTOR signalling pathway in LN229 cells (Figure 4C; Supplementary Figure S1C). We also demonstrated that the AKT inhibitor MK2206 can block the CBX2-activated AKT/mTOR signalling pathway (Figure 4D; Supplementary Figure S1D). In addition, MK2206 treatment inhibited the viability, proliferation, and chemoresistance of CBX2-overexpression cells (Figures 4E–K). These findings suggest that CBX2 promotes cell proliferation and chemoresistance by inhibiting PTEN expression and activating the AKT/mTOR signalling pathway.

### The impact of CBX2 on the tumorigenicity and TMZ chemoresistance of glioma cells *in vivo*

**FIGURE 5:**
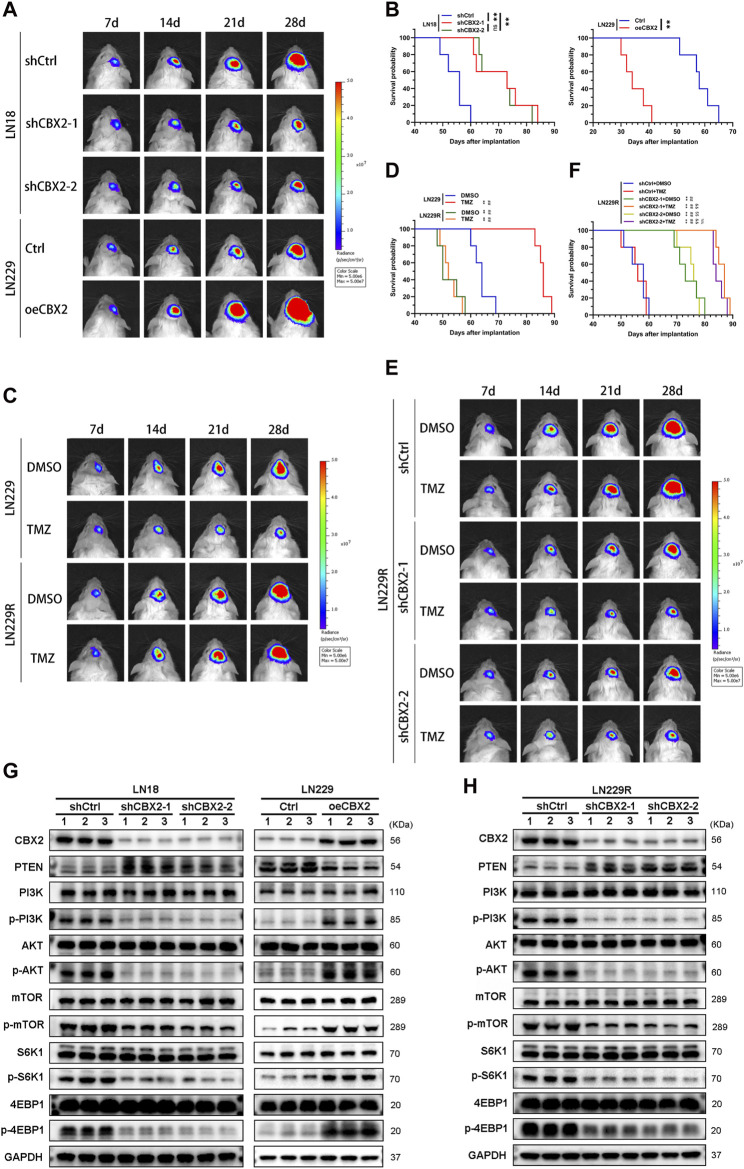
The impact of CBX2 on the tumorigenicity and TMZ chemoresistance of glioma cells *in vivo*. **(A)** Pseudocolored bioluminescence images depict orthotopic tumors originating from CBX2-knockdown LN18 cells and CBX2-overexpression LN229 cells, with each group comprising 5 mice. **(B)** Survival curves display the outcomes of xenografted mice derived from CBX2-knockdown LN18 cells and CBX2-overexpressing LN229 cells (**p < 0.01, ns: not significant). **(C)** Pseudocolored bioluminescence images portray orthotopic tumors arising from LN229/LN229R cells, both with/without TMZ treatment, in groups of 5 mice each. **(D)** Survival curves of xenografted mice originating from LN229/LN229R cells with/without TMZ treatment are presented (**p < 0.01, compared with LN229 + DMSO; ##*p* < 0.01, compared with LN229 + TMZ). **(E)** Pseudocolored bioluminescence images depict orthotopic tumors derived from CBX2-knockdown LN229R cells, with/without TMZ treatment, in groups of 5 mice each. **(F)** Survival curves of xenografted mice arising from CBX2-knockdown LN229R cells, with/without TMZ treatment, are displayed (**p < 0.01, compared with shCtrl + DMSO; ##*p* < 0.01, compared with shCtrl + TMZ; &&*p* < 0.01, compared with shCBX2-1 + DMSO; $$*p* < 0.01, compared with shCBX2-1+TMZ; %%*p* < 0.01, compared with shCBX2-2+ DMSO). **(G)** WB analysis was conducted to assess the expression of CBX2 and proteins associated with the PTEN/AKT/mTOR pathway in CBX2-knockdown LN18 cells and CBX2-overexpressing LN229 cells. **(H)** WB was also performed to evaluate the expression of CBX2 and proteins linked to the PTEN/AKT/mTOR pathway in CBX2-knockdown LN229R cells.

To assess the impact of CBX2 on tumorigenicity and TMZ resistance of glioma cells *in vivo*, we established a mouse model of intracranial xenograft tumors. At 7 days after implantation, there was no significant difference in fluorescence intensity between the groups. Over time, the fluorescence values in the shCBX2-1 and shCBX2-2 groups were significantly lower than those in the shCtrl group, being approximately 50% at 14 days, 25% at 21 days, and 20% at 28 days. However, the fluorescence intensity in the oeCBX2 group was 3, 5, and 5 times higher than that of the Ctrl group at 14 days, 21 days, and 28 days, respectively (Figure 5A; Supplementary Figure S1E). Kaplan-Meier analysis further demonstrated that knockdown/overexpression of CBX2 prolonged/shortened the survival time of xenografted mice (Figure 5B). Furthermore, we successfully established a TMZ resistant cell line, LN229R, in which the IC50 value increased more than fivefold, concomitant with an elevated expression of CBX2 (Supplementary Figures S1F, G). *In vivo* experiments, we observed that in the LN229R group, whether treated with TMZ or not, fluorescence intensity remained consistently high. In contrast, in the LN229 group, TMZ treatment significantly inhibited the growth of gliomas, with fluorescence intensity at 28 days being 10% of the DMSO group, corroborating that TMZ effectively inhibited the growth of LN229 cells (Figure 5C; Supplementary Figure S1H), resulting in longer survival time (Figure 5D). However, TMZ exhibited no inhibitory effect on LN229R cells, failing to improve the survival time of mice (Figures 5C, D; Supplementary Figure S1H). Subsequently, we implemented CBX2-knockdown LN229R, and the results demonstrated that CBX2 knockdown not only slowed glioma growth and prolonged survival without TMZ treatment but also significantly enhanced tumor suppression and extended survival when combined with TMZ treatment. At 28 days, the fluorescence intensity of TMZ-treated CBX2-knockdown LN229R group was only 10% of the control group. (Figures 5E, F; Supplementary Figure S1I). These findings collectively underscore that CBX2 knockdown not only curtails tumor growth but also mitigates TMZ chemoresistance. Additionally, we substantiated these effects through WB, which revealed that CBX2 knockdown or overexpression modulated PTEN expression and impacted the activation of proteins associated with the AKT/mTOR pathway in mice (Figure 5G; Supplementary Figure S1J). Moreover, CBX2 knockdown in LN229R promoted PTEN expression and hindered the activation of proteins linked to the AKT/mTOR pathway (Figure 5H; Supplementary Figure S1K).

### CBX2 regulates PTEN expression through modulation of H3K27me3

**FIGURE 6:**
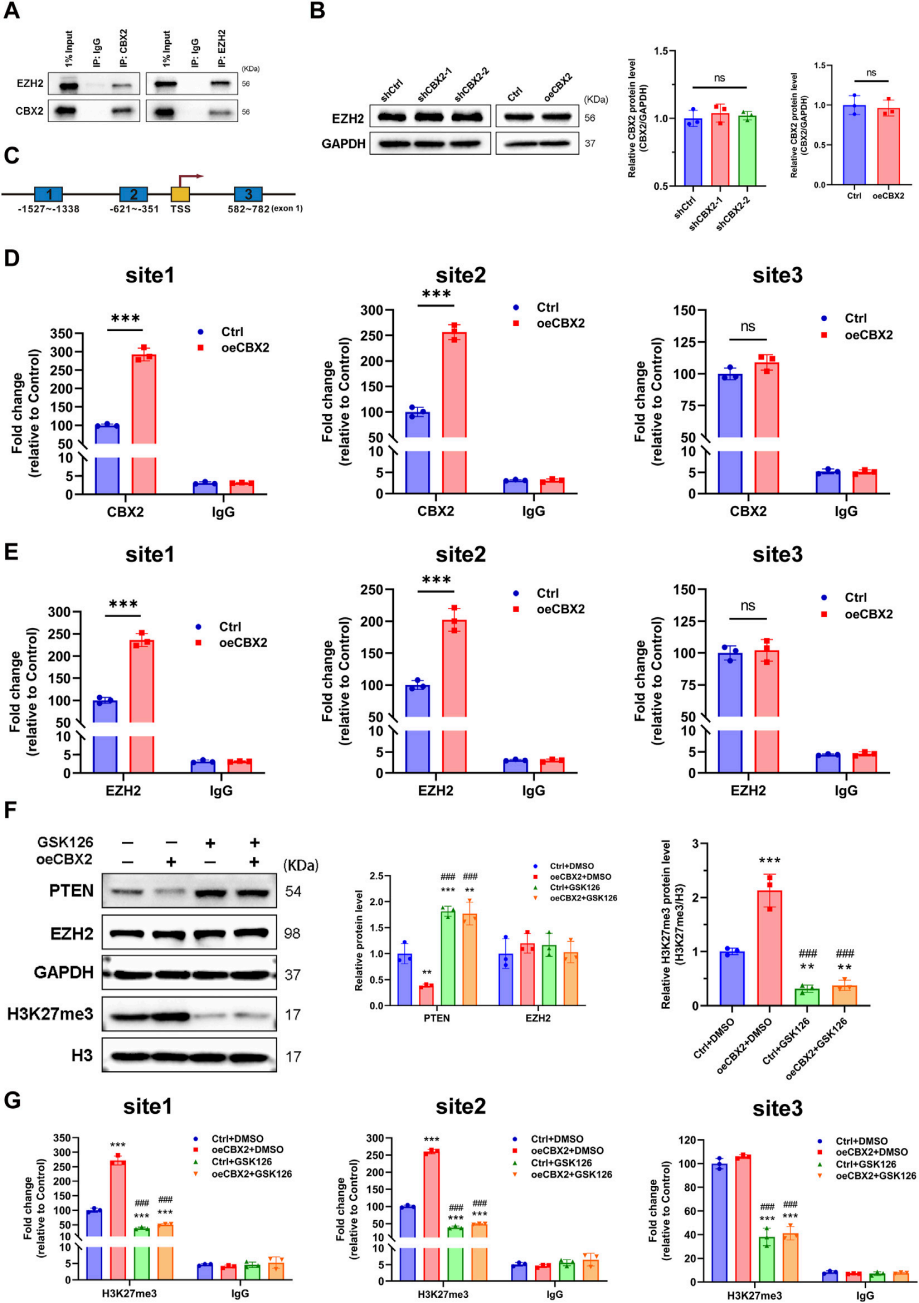
CBX2 regulates PTEN expression through H3K27me3. **(A)** CBX2 interacts with EZH2. Co-IP was performed in LN229 cells with anti-EZH2 or anti-CBX2 antibodies. **(B)** The levels of EZH2 were assessed in CBX2-knockdown LN18 cells and CBX2-overexpression LN229 cells. **(C)** Schematic presentation of three regions relative to the *PTEN* promotor site used as primers for the ChIP assay. **(D)** CBX2 occupancy in the *PTEN* promoter region (site 1, site 2 and site 3). IgG was used as a negative control. **(E)** EZH2 occupancy in the *PTEN* promoter region (site 1, site 2 and site 3). IgG was used as a negative control (***p < 0.001; ns: not significant). **(F)** LN229 cells overexpressing CBX2 were treated with the EZH2 inhibitor GSK126 (10 μM) for an additional 24 h, and then the cells were harvested and subjected to WB (**p < 0.01, ***p < 0.001, compared with Ctrl + DMSO; ###*p* < 0.001, compared with oeCBX2 + DMSO). **(G)** ChIP was performed to assess H3K27me3 occupancy in the *PTEN* promoter region (site 1, site 2 and site 3). IgG was used as a negative control (***p < 0.001, compared with Ctrl + DMSO; ###*p* < 0.001, compared with oeCBX2 + DMSO).

**FIGURE 7:**
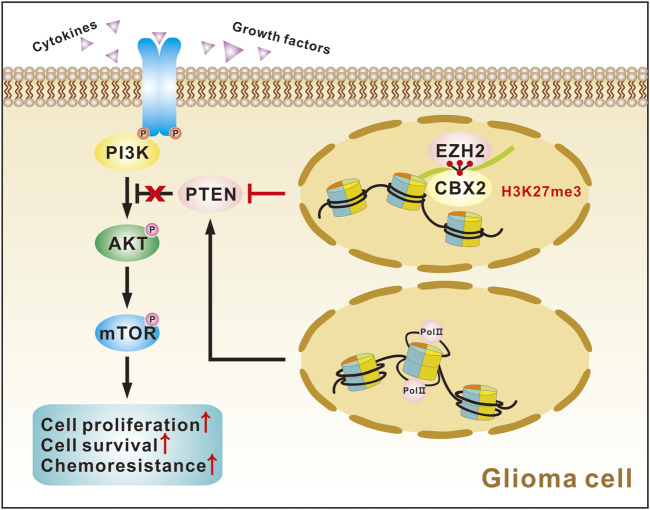
Graph showing CBX2 function in glioma cells.

Finally, we investigated how CBX2 regulates PTEN expression at the transcriptional level. Previous studies have shown that CBX2 and EZH2 have synergistic effects and are often involved in the epigenetic regulation of chromatin (Hu et al., 2022; Xu et al., 2023). We performed co-IP experiments and proved the binding of CBX2 and EZH2 in glioma cells (Figure 6A). However, CBX2 cannot regulate the expression of EZH2 (Figure 6B). Using ChIP assay, we determined the core interaction region of CBX2 on the *PTEN* promoter: the −1,527–−1,338 region and the −621–−351 region (Figures 6C, D). Meanwhile, we observed that the overexpression of CBX2 made the binding of EZH2 and *PTEN* promoter closer (Figure 6E). Because EZH2 acts as a histone methylase, we assessed the H3K27me3 modification level of the *PTEN* gene in glioma cells. GSK126 was also used to inhibit EZH2 activity, and WB showed that GSK126 decreased the level of the EZH2-specific methylation marker H3K27me3, while the levels of total histone H3 and EZH2 did not change (Figure 6F). The ChIP assay demonstrated that overexpression of CBX2 increased H3K27me3 levels in the *PTEN* promoter region, and this effect was reversed by the EZH2 inhibitor GSK126 (Figure 6G). These results clearly suggest that CBX2 induces transcriptional inactivation of PTEN by recruiting EZH2 to regulate the H3K27me3 level of the *PTEN* promoter (Figure 7).

## Discussion

Glioma is a highly prevalent intracranial tumour with resistance to multiple therapies and poor prognosis. Unfortunately, the pathogenesis of glioma remains a mystery and may be influenced by a complex network of genes, signalling pathways and molecular components.

Recent studies have shown that the expression of CBX2 is closely related to the occurrence and development of osteosarcoma (Han et al., 2019), ovarian cancer (Wheeler et al., 2018), breast cancer (Chen et al., 2017), liver cancer (Mao et al., 2019) and prostate cancer (Clermont et al., 2016). Zheng et al. (2019) found that CBX2 is highly expressed in breast cancer and is closely related to a poor prognosis in patients. Piqué et al. (2019) confirmed that CBX2 is also an oncogene in breast cancer. Knocking down CBX2 inhibits the proliferation and invasion of breast cancer cells *in vivo* and *in vitro* (Zheng et al., 2019). Mao et al. (2019) found that the high expression of CBX2 is related to the poor prognosis of patients with hepatocellular carcinoma. CBX2 knockdown inhibits the proliferation and promotes the apoptosis of hepatocellular carcinoma cells. The underlying mechanism is that CBX2 knockdown inhibits the expression of WT1 interacting protein, stimulates the Hippo pathway, and leads to phosphorylation inactivation of YAP. Compared with that in normal gastric cells, CBX2 expression is increased in gastric cancer cell lines. CBX2 knockdown inhibits the nuclear cytoplasmic translocation of YAP, induces its phosphorylation, and inhibits the activation of the β-catenin signalling pathway in gastric cancer (Zeng et al., 2021). Other investigations found that CBX2 interacts with noncoding RNAs and regulates tumorigenesis, progression and chemoresistance in multiple cancers, including pancreatic adenocarcinoma, ovarian cancer and osteosarcoma (Han et al., 2019; Dou et al., 2020; Wang et al., 2020; Zhu et al., 2021).

In the current study, through bioinformatics analysis, we found that CBX2 is upregulated in gliomas and is closely related to pathological grade and a poor prognosis, which was verified by IHC analysis of clinical samples. In addition, we found that CBX2 is closely related to glioma chemoresistance, and tumorigenesis studies *in vivo* also strongly support its role in glioma progression and chemoresistance. In their latest study, Wang et al. (2021) also showed that CBX2 induces the proliferation and invasion of glioma cells through the PI3K/AKT pathway, which is consistent with our results.

In the current study, the molecular mechanism investigation showed that CBX2 activates the AKT/mTOR signalling pathway by inhibiting the expression of PTEN and affects the carcinogenicity and chemoresistance of glioma cells. The AKT/mTOR signalling pathway is one of the most critical signal transduction pathways in cellular activities and participates in the regulation of many important biological processes, such as blocking apoptosis and promoting tumorigenesis, development and chemoesistance (Davis et al., 2014; Wei et al., 2020; Tang et al., 2022). PTEN, a tumour suppressor, is a dual phosphatase. Its main function is to dephosphorylate phosphatidylinositol 3, 4, 5-trisphosphate to phosphatidylinositol 4, 5-diphosphate, antagonizing the PI3K/AKT signal (Blanco-Aparicio et al., 2007). Herein, we found that the downregulation of CBX2 significantly increased the expression of PTEN and decreased the phosphorylation of AKT and mTOR, and conversely, upregulation of CBX2 significantly reversed this process. When the phosphorylation of AKT was disturbed, the effect of CBX2 on glioma proliferation and chemoresistance was abolished, suggesting that CBX2 promotes glioma progression and chemoresistance through PTEN-controlled AKT activation. Similarly, the PTEN/AKT/mTOR pathway has been reported to be closely related to cell proliferation and chemoresistance in a variety of tumours (Wang et al., 2016; Wu et al., 2016a; Wu et al., 2016b).

Histone methylation is an important process of chromatin modification and is regulated by a variety of histone-modifying enzymes. Histone methylation participates in many cellular processes, such as DNA repair, DNA replication and gene transcription (Casas-Delucchi et al., 2012; Gonzalez and Li, 2012; Yang et al., 2019). The transcription of PTEN is controlled by the level of histone methylation in the promoter region mediated by histone modifying enzymes (Jarome et al., 2018; Kim et al., 2021). Abnormal transcription of PTEN is involved in the occurrence and development of many kinds of tumours (Tang et al., 2015; Lei et al., 2016; Wang et al., 2019). Wang et al. (2019) reported that the histone modifying enzyme JARID2 promotes the progression of bladder cancer through H3K27me3-mediated downregulation of PTEN and hyperactivation of AKT. JARID1B promotes the metastasis and epithelial-mesenchymal differentiation of hepatocellular carcinoma cells by enhancing trimethylation of histone H3 lysine 4 on the *PTEN* promoter and thereby mediates PTEN transcriptional inactivation and AKT activation (Tang et al., 2015). Similarly, EZH2 is a catalytic subunit of PRC2 and acts as a histone methyltransferase that catalyses the H3K27me3 to induce chromosome condensation, thereby inhibiting the transcription of target genes (Marchesi and Bagella, 2016).

An increase in H3K27me3 level of the *PTEN* promoter region mediated by EZH2 increases the transcription of the silenced *PTEN* gene and activates the AKT/mTOR pathway (Jarome et al., 2018)^.^ CBX2 mainly targets PRC1 to chromatin, and the C-terminal multi-comb receptor box of CBX2 can specifically recognize H3K27me3, thus enhancing the inhibition of gene expression (Ma et al., 2014). The latest research shows that CBX2 and EZH2 have synergistic tumorigenic effects (Hu et al., 2022; Xu et al., 2023). In this study, we proved the binding of CBX2 and EZH2 in glioma. We also demonstrated that overexpression of CBX2 led to enrichment of CBX2 in the *PTEN* promoter region and thus increased the H3K27me3 level on the *PTEN* promoter, and this effect could be blocked by using an EZH2 inhibitor, indicating that CBX2 is necessary for the H3K27me3 modification of *PTEN*. Taken together, these findings confirm that CBX2 inhibits the expression of PTEN by recruiting EZH2 and altering the H3K27me3 level on the *PTEN* promoter.

## Conclusion

In summary, we found that CBX2 is overexpressed in gliomas and is associated with high tumor grade, TMZ chemoresistance, and poor prognosis in glioma patients. Examining the potential molecular mechanism, we found that CBX2 inhibits PTEN transcription by recruiting EZH2 to regulate the H3K27me3 level of the *PTEN* promoter and thus activates the AKT/mTOR pathway to accelerate glioma progression and TMZ chemoresistance. Pharmacological targeting CBX2 could provide a novel therapeutic approach for the treatment of gliomas.

## Data Availability

The datasets presented in this study can be found in online repositories. The names of the repository/repositories and accession number(s) can be found in the article/Supplementary Material.

## Supplementary material

SUPPLEMENTARY FIGURE S1
**(A)** Knockdown of CBX2 in LN18 cells. LN18 cells were transduced with three shRNAs targeting human CBX2 or a control shRNA using lentivirus. Subsequently, WB was conducted to analyze the protein expression level of CBX2 (****p* < 0.001, compared with shCtrl; #*p* < 0.05, compared with shCBX2-0). **(B)** Overexpression of CBX2 in LN229 cells. LN229 cells were transduced with human CBX2 using lentivirus. WB was then performed to assess the protein levels of CBX2 (****p* < 0.001). **(C)** Expression level of relative protein in LN18 cells transfected with CBX2 shRNAs (****p* < 0.001, compared with shCtrl) and LN229 cells transduced with human CBX2 via lentivirus (***p* < 0.01, ****p* < 0.001, compared with Ctrl). **(D)** Expression level of relative protein in CBX2-overexpression LN229 cells treated with the AKT inhibitor MK2206 (**p* < 0.05, ***p* < 0.01, ****p* < 0.001; ns: not significant). **(E)** Quantification of bioluminescence signals in orthotopic tumors originating from mice implanted with CBX2-knockdown LN18 cells and CBX2-overexpression LN229 cells. **(F)** Expression level of CBX2 protein in LN229/LN229R cells (***p* < 0.01). **(G)** LN229/LN229R cells were subjected to TMZ treatment, and cell viability was determined using CCK-8 assay (****p* < 0.001). **(H)** Quantification of bioluminescence signals in orthotopic tumors originating from mice implanted with LN229/LN229R cells with/without TMZ treatment (****p* < 0.001, compared with LN229+DMSO; ###*p* < 0.001, compared with LN229+TMZ). **(I)** Quantification of bioluminescence signals in orthotopic tumors originating from mice implanted with LN229R/CBX2-knockdown LN229R cells with/without TMZ treatment (****p* < 0.001, compared with shCtrl+DMSO; ###*p* < 0.001, compared with shCtrl+TMZ; &&&*p* < 0.001, compared with shCBX2-1+DMSO; $$$p<0.001, compared with shCBX2-1+TMZ; %%%*p* < 0.001, compared with shCBX2-2+DMSO). **(J)** Expression level of CBX2 and proteins associated with the PTEN/AKT/mTOR pathway in CBX2-knockdown LN18 cells (****p* < 0.001, compared with shCtrl) and CBX2-overexpression LN229 cells (****p* < 0.001, compared with Ctrl). **(K)** Expression level of CBX2 and proteins linked to the PTEN/AKT/mTOR pathway in CBX2-knockdown LN229R cells (****p* < 0.001, compared with shCtrl).
