# Supplementary material for: CBX2 enhances the progression and TMZ chemoresistance of glioma via EZH2-mediated epigenetic silencing of PTEN expression
Source: Front Pharmacol. 2024 Jul 24;15:1430891. doi: 10.3389/fphar.2024.1430891 (PMC11303140; doi:10.3389/fphar.2024.1430891)
Supplement: Supplementary file 1 [file Table1.docx]

**Supplementary Table S1.** shRNAs and primer sequences

| Gene | Sequence (5’ – 3’) |
| --- | --- |
| CBX2 shRNAs |  |
| 0 | GGCCTTCCAGAAGAAGGAACA |
| 1 | GCTGGTCCTCCAAACATAACA |
| 2 | GCTCCCTCCAAATCCAAGTCC |
| shCtrl | GCAGCAACTGGACACGTGATCTTAA |
| *PTEN* | Forward primer: TGGATTCGACTTAGACTTGACCT |
|  | Reverse primer: GGTGGGTTATGGTCTTCAAAAGG |
| *GAPDH* | Forward primer: TGACAACTTTGGTATCGTGGAAGG |
|  | Reverse primer: AGGCAGGGATGATGTTCTGGAGAG |
| *PTEN* promotor Site 1 | Forward primer: CAGAATAGGTCGATGTAGAGC |
|  | Reverse primer: GGGAACTGGTTACACAAGCAC |
| *PTEN* promotor Site 2 | Forward primer: GGATGAGGTGATACACGCTG |
|  | Reverse primer: GACCACGCTGCTCAGTGTAG |
| *PTEN* promotor Site 3 | Forward primer: TCGCCTGTCACCATTTCCAG |
|  | Reverse primer: AGACGAATAATCCTCCGAACG |
