# Supplementary figures and images for: CBX2 enhances the progression and TMZ chemoresistance of glioma via EZH2-mediated epigenetic silencing of PTEN expression
Source: Front Pharmacol. 2024 Jul 24;15:1430891. doi: 10.3389/fphar.2024.1430891 (PMC11303140; doi:10.3389/fphar.2024.1430891)

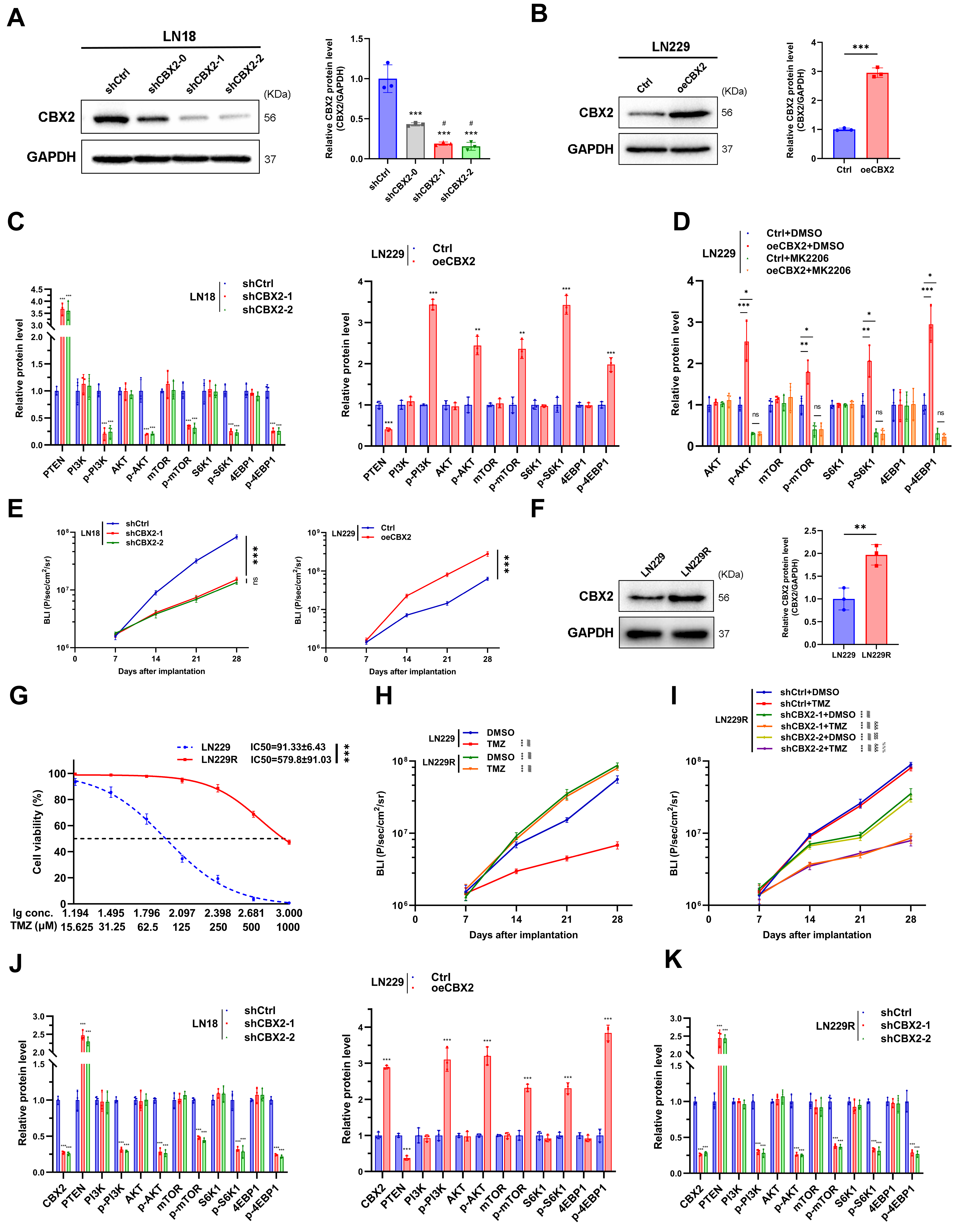

Supplement: Supplementary file 2 [file Image1.TIF]
