# Supplementary material for: CBX2 enhances the progression and TMZ chemoresistance of glioma via EZH2-mediated epigenetic silencing of PTEN expression
Source: Front Pharmacol. 2024 Jul 24;15:1430891. doi: 10.3389/fphar.2024.1430891 (PMC11303140; doi:10.3389/fphar.2024.1430891)
Supplement: Supplementary file 3 [file Table2.docx]

**Supplementary Table S2.** The relevant information of the antibodies.

| Markers | Antibody Names | Supplier | Catalog No. | Application | Dilution |
| --- | --- | --- | --- | --- | --- |
| CBX2 | Anti-CBX2 antibody | Abcam | ab235305 | IHCWB | 1:200  1:1000 |
| CBX2 | Anti-CBX2 antibody | Novus | NBP1-40342 | co-IP  ChIP | 1:100  1:100 |
| PTEN | PTEN (D4.3) XP^®^ Rabbit mAb | CST | #9188 | WB | 1:1000 |
| PI3K | PI3 Kinase p110α (C73F8) Rabbit mAb | CST | #4249 | WB | 1:1000 |
| p-PI3K | Phospho-PI3 Kinase p85 (Tyr458)/p55 (Tyr199) Antibody | CST | #4228 | WB | 1:1000 |
| AKT | Akt (pan) (C67E7) Rabbit mAb | CST | #4691 | WB | 1:1000 |
| p-AKT | Phospho-Akt (Ser473) (D9E) XP^®^ Rabbit mAb | CST | #4060 | WB | 1:2000 |
| mTOR | mTOR (7C10) Rabbit mAb | CST | #2983 | WB | 1:1000 |
| p-mTOR | Phospho-mTOR (Ser2448) (D9C2) XP^®^ Rabbit mAb | CST | #5536 | WB | 1:1000 |
| S6K1 | p70 S6 Kinase (49D7) Rabbit mAb | CST | #2708 | WB | 1:1000 |
| p-S6K1 | Phospho-p70 S6 Kinase (Thr389) (108D2) Rabbit mAb | CST | #9234 | WB | 1:1000 |
| 4EBP1 | 4E-BP1 (53H11) Rabbit mAb | CST | #9644 | WB | 1:1000 |
| p-4EBP1 | Phospho-4E-BP1 (Thr37/46) (236B4) Rabbit mAb | CST | #2855 | WB | 1:1000 |
| GAPDH | GAPDH (D16H11) XP^®^ Rabbit mAb | CST | #5174 | WB | 1:1000 |
| H3K27me3 | Tri-Methyl-Histone H3 (Lys27) (C36B11) Rabbit mAb | CST | #9733 | WB  ChIP | 1:1000  1:50 |
| H3 | Histone H3 (D1H2) XP^®^ Rabbit mAb | CST | #4499 | WB | 1:2000 |
| EZH2 | Ezh2 (D2C9) XP^®^ Rabbit mAb | CST | #5246 | WB  co-IP  ChIP | 1:1000  1:300  1:100 |
